# Perspectives in Therapy of Chronic Rhinosinusitis

**DOI:** 10.3390/diagnostics12102301

**Published:** 2022-09-23

**Authors:** Jacek Brzost, Katarzyna Czerwaty, Karolina Dżaman, Nils Ludwig, Katarzyna Piszczatowska, Mirosław J. Szczepański

**Affiliations:** 1 The Children’s Memorial Health Institute, 04-730 Warsaw, Poland; 2 Department of Otolaryngology, The Medical Centre of Postgraduate Education, 01-813 Warsaw, Poland; 3 Department of Oral and Maxillofacial Surgery, University Hospital Regensburg, 93053 Regensburg, Germany; 4 Department of Biochemistry, Medical University of Warsaw, 02-097 Warsaw, Poland

**Keywords:** chronic rhinosinusitis, sinusitis, treatment, biologics

## Abstract

The recent classification of chronic rhinosinusitis (CRS) focusses on investigating underlying immunopathophysiological mechanisms. Primary CRS is subdivided based on endotype dominance into type 2 (that relates mostly to the Th2 immune response with high levels of IL-5, IL-13, and IgE), or non-type 2 (that corresponds to the mix of type 1 and type 3). The treatment selection of CRS is dependent on endotype dominance. Currently, the majority of patients receive standardized care—traditional pharmacological methods including local or systemic corticosteroids, nasal irrigations or antibiotics (for a selected group of patients). If well-conducted drug therapy fails, endoscopic sinus surgery is conducted. Aspirin treatment after aspirin desensitization (ATAD) with oral aspirin is an option for the treatment in nonsteroidal anti-inflammatory drug (NSAID)-exacerbated respiratory disease (N-ERD) patients. However, in this review the focus is on the role of biological treatment—monoclonal antibodies directed through the specific type 2 immune response targets. In addition, potential targets to immunotherapy in CRS are presented. Hopefully, effective diagnostic and therapeutic solutions, tailored to the individual patient, will be widely available very soon.

## 1. Introduction

Chronic rhinosinusitis (CRS), characterized by persistent inflammation of the sinonasal mucosa (SM), is one of the most common health care problems affecting 5 to 12% of the western population [1,2]. Among CRS patients, asthma is one of the most frequent comorbidities. The multicentre Global Allergy and Asthma European Network study showed that around 20% of CRSsNP patients and almost 50% of CRSwNP patients reported asthma [3]. Additionally, a correlation between the severity of asthma and the clinical and radiological features of CRS was observed [4]. There is also a relationship between allergy and specific endotypes of CRS, such as allergic fungal rhinosinusitis (AFRS) and central compartment allergic disease (CCAD) [5]. Overall, CRS negatively affects patients’ quality of life (QoL) [6,7] and productivity [8]. This review focuses on the current state of knowledge with regards to treatment methods in CRS taking into account the new classification of CRS with particular emphasis on new biological treatment and other promising directions of research in this area.

## 2. New Classification of CRS

CRS is frequently divided into two clinical phenotypes based on the presence of endoscopically visualized polyps in the middle nasal meatus: CRS with nasal polyps (CRSwNP) and CRS without nasal polyps (CRSsNP) [9]. Nasal polyps are defined as inflammatory lesions that originate from the ethmoid sinus and project into the nasal airway [10]. This classification is simple to use and practical for clinicians, but does not provide information about pathophysiologic mechanisms and, as a result, does not allow for the selection of the optimal treatment. Dividing into endotypes based on the inflammatory profiles is not as simple as the endoscopic determination of polyps, but is more relevant in relating to the diagnosis and specific treatment strategy for the different subgroups of CRS patients [11]. In CRSwNP, sinonasal tissue displays significantly higher levels of eosinophilic markers and Th2 polarization with high IL-5, IL-13, and IgE concentrations, whereas CRSsNP is characterized by a Th1 polarization with high levels of IFN-γ and TGF-β [12].

**Figure 1:**
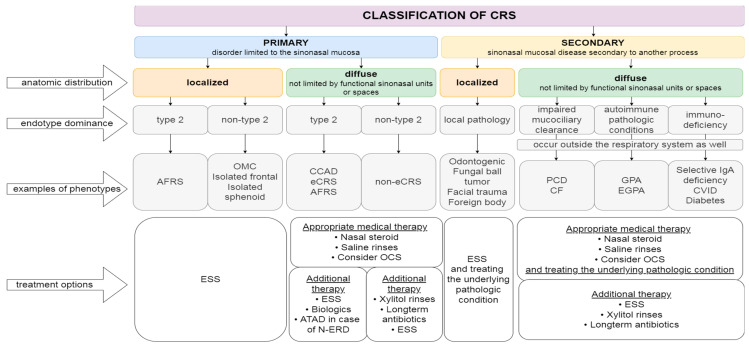
Classification of CRS created by Grayson et al. with general information about appropriate treatment for particular endotypes ([2,18], modified). AFRS, allergic fungal rhinosinusitis; OMC, ostiomeatal complex; CCAD, central compartment atopic disease; eCRS, eosinophilic CRS; PCD, primary ciliary dyskinesia; CF, cystic fibrosis; GPA, granulomatosis with polyangiitis; EGPA, eosinophilic granulomatosis with polyangiitis; CVID, common variable immunodeficiency; OCS, oral corticosteroid; ESS, endoscopic sinus surgery; ATAD, aspirin treatment after desensitization; N-ERD, non-steroidal anti-inflammatory drugs-exacerbated respiratory disease.

The European Position Paper on Rhinosinusitis and Nasal Polyps 2020 (EPOS 2020) steering group proposed a new division of CRS (Figure 1). A new classification system distinguishes primary and secondary CRS. Further divisions are based on the anatomic distribution (localized or diffuse disease), endotype dominance (understanding the underlying pathophysiology in association with raised IgE, IL-5, eosinophilia, and periostin), and clinical phenotypes [2,13]. Primary CRS is divided into localized (typically unilateral) and diffuse (not limited by functional sinonasal units or spaces). A further division is based on endotype dominance into type 2, that relates mostly to the Th2 immune response, or non-type 2, which corresponds to the mix of type 1 and type 3 inflammation. In a group of localized primary CRS, two phenotypes are distinguished: AFRS and isolated sinusitis. Clinically diffuse primary CRS are subdivided into eosinophilic chronic rhinosinusitis (eCRS), AFRS and central compartment allergic disease (CCAD) or non-eosinophilic chronic rhinosinusitis (non-eCRS) [2,13].

**Figure 2:**
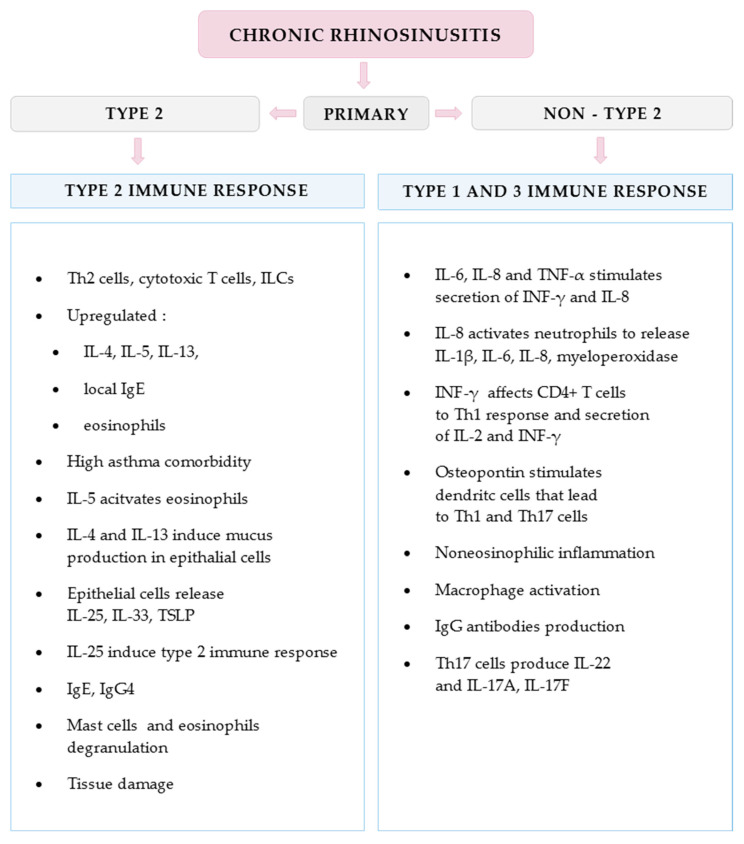
Selected immune activity mechanisms involved in type 2 and non-type 2 immune response in case of chronic rhinosinusitis described by Grayson et al. ([18], modified).

In the past, CRSsNP was considered to mainly promote the Th1 immune response pathway, CRSwNP—Th2 (Figure 2). It is now known that patients often present more than one type of inflammatory endotype. In the US and Europe, the most frequent endotype in CRSsNP is type 2, as opposed to type 1 or type 3 [14]. Studies have shown that T cells obtained from European patients with CRSwNP were Th2-dependent while tending towards neutrophilic inflammation in the Asian population [15,16]. The presence of three main inflammatory endotypes found in ethmoid tissues and nasal lavage fluid of CRSsNP patients have promising diagnostic and therapeutic potential [17].

Typical for eosinophilic CRS (e-CRS) is tissue and systemic eosinophilia, whereas non-eCRS are characterized by lack of tissue eosinophilia [18]. Patients with non-eCRS are often older, more resistant to corticosteroid treatment and less frequently complain of smell loss [13]. CCAD is an IgE-associated disorder that may have a high association with inhalant allergy and is defined by polypoid changes of the entire central sinonasal compartment, while the lateral SM remains relatively normal [18,19]. After ESS in CCAD patients, rates of polyp recurrence and revision ESS are significantly lower compared to patients with other CRSwNP subtypes [20]. To differentiate diffuse disease, causal mechanisms should be taken into consideration. In the course of CRS, 23% or almost 50% in other reports of patients recalcitrant to treatment or 13% of recurrency are due to immunoglobulin deficiencies and mostly it relates to common variable immunodeficiency (CVID) and IgA deficiency [21,22,23]. In CRS interaction between environmental factors and the host immune system is disrupted. The affected SM develops three main kinds of defensive reactions that lead to tissue remodelling (polyp formation, goblet cell hyperplasia, and epithelial barrier abnormalities) and clinical symptoms.

Among all the variety of bacteria, Staphylococcus aureus (SA) deserves special attention. This kind of bacteria contributes to nasal polyp formation, prolonged tissue inflammation, and bacterial dysbiosis [24]. SA endotoxins can induce IgE production that can elicit the inflammatory cascade [25] and directly increase the expression of IL-33, TSLP, IL-5, and IL-13 in NP tissue and elevate expression levels of TSLP and IL-33 receptors [26]. SA infection is particularly relevant in CRSwNP. SA is a strong biofilm producer and can secrete toxins to injure leukocidins, which may have the ability to cause an impairment of local host immunity against SA through injuring immune cell types participating in innate and adaptive immune responses [27].

Air pollution is one of the major troubles and challenges, notably in big city agglomerations, which leads to the severity of CRS symptoms, especially in the group of CRS without nasal polyps (CRSsNP) patients exposed to air pollutants [28]. A higher degree of tissue inflammation is associated with increased ozone exposure [29]. CRS is more common in smokers [30], and the burden of CRS symptoms is associated with active cigarette smoking [31]. Exposure to different occupational agents is associated with the onset of CRS [32,33] and an increased need for endoscopic sinus surgery in CRS patients [34,35].

## 3. Type 2 and Non-Type 2 Chronic Rhinosinusitis

**Figure 3:**
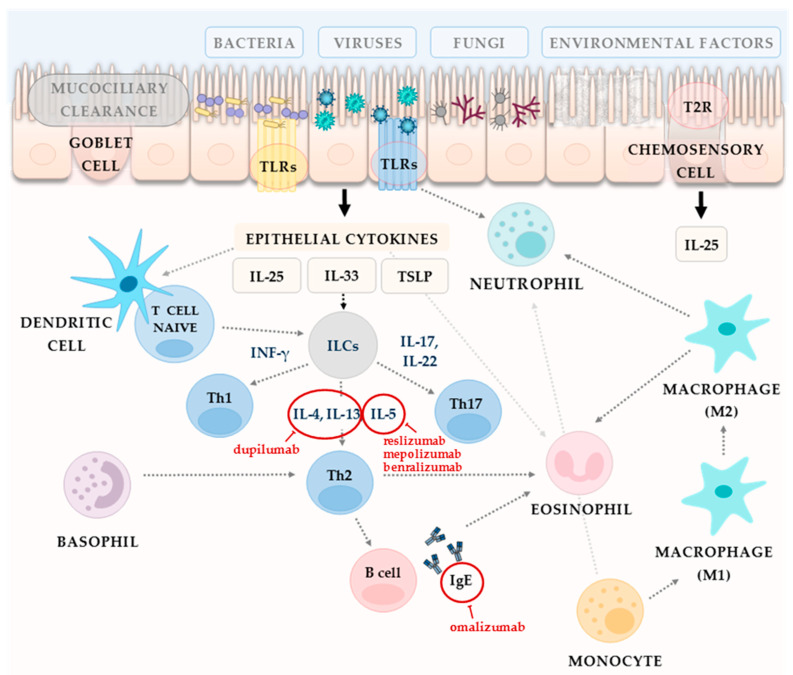
General immune mechanism involved in the pathogenesis of CRS and targets for selected biologics. Immune response combines the airway epithelium response as a first line of defence in sinuses that meet environmental factors with innate immune activity which leads to adaptive immune activation ([2,13], modified).

Understanding the underlying immunological pathophysiology, they distinguished two dominant endotypes: type 2, related mostly to the Th2 immune response, and non-2-type (Figure 2 and Figure 3) [2,13].

### 3.1. Type 2 Inflammatory Response

The type 2 immune response pathway is initiated by epithelial cell-derived cytokines, such as IL-25, IL-33, and thymic stromal lymphopoietin (TSLP), which are released after exposure to harmful environmental factors and activate innate lymphoid cells (ILCs) [36]. ILC2 and IL-25 are significantly enriched in SM of CRSwNP patients [37,38]. Type 2 is characterized by upregulated production of the cytokines IL-4, IL-5, and IL-13, local IgE and profound eosinophilia [13].

Type 2 endotype in CRS is associated with the presence of NPs, asthma comorbidity, smell loss and allergic mucin [14] and also are less likely to respond to current treatment, presenting more frequent recurrences as opposed to pure type 1 or 3 endotypes [2]. In type 2 CRS, the main complaints are smell loss or blockage/congestion, and a greater tendency towards recurrence is observed [2,39,40].

### 3.2. Non-Type 2 Inflammatory Response

The term “non-type 2” covers a mix of type 1 and type 3 inflammatory responses. In both pathways, the epithelial response to environmental stimuli leads to stimulation of dendritic cells and thereby Th1- and Th17-cell differentiation, which leads to non-eosinophilic inflammation. Type 1 immune response is directed against intracellular bacteria, protozoa and viruses through the production of IFN-γ [41]. Type 3 cell mediated immunity defence against extracellular bacteria and fungi through the production of IL-17 alone or in combination with IL-22 [41].

## 4. Conventional Treatment Approaches

The new approach to treatment takes into consideration pathophysiology and causal mechanisms (Figure 1). Nowadays, in order to establish endotype dominance and to choose the most effective treatment, phenotype of CRS, markers (eosinophils, periostin, IgE in blood or tissue), and treatment response should be considered [2]. Tissue and serum periostin levels appear elevated in CRSwNP, especially in eosinophilic inflammation, compared to CRSsNP and controls [42].

### 4.1. Appropriate Pharmacological Medical Therapy

The main pharmacological treatment methods used for years are saline irrigation, topical and systemic steroids and antibiotics. In certain groups of patients, leukotriene modifiers or aspirin treatment after aspirin desensitization (ATAD) can be applied.

#### 4.1.1. Nasal Irrigations

Nasal irrigations with isotonic saline or Ringer’s lactate are recommended as first line therapies in CRS, and the addition of xylitol, sodium hyaluronate, and xyloglucan may have a positive effect [2]. In adults with CRS, large-volume devices (≥60 mL) are effective and buffered isotonic saline is more tolerable than non-buffered or hypertonic saline [43].

#### 4.1.2. Topical Steroids

Topical steroids are the first-line treatment in diffuse CRS [2]. The effectiveness and safety of intranasal corticosteroids (INCS) in different endotypes of CRS, also pre- and post-operatively, have been confirmed in many studies [44,45,46,47,48,49,50,51,52,53,54,55,56,57,58]. There are a few new methods of delivering steroids into the sinuses: irrigations (budesonide and mometasone), resolve steroid-eluting nasal stents, exhalation delivery system fluticasone device [59]. In a randomized, double-blind study the effect of nasal mometasone irrigation or simple mometasone nasal spray in CRS patients after ESS was confirmed [58]. In clinical trials with budesonide added to large-volume, low pressure saline sinus irrigation improvement of sino-nasal symptoms and endoscopic scores was demonstrated [60]. Nevertheless, in a group of post-ESS CRS patients, nasal saline alone and with the addition of budesonide improved QoL, but neither intervention was significantly more effective [61]. In selected patients using budesonide, nasal irrigation asymptomatic hypothalamic-pituitary-adrenal axis suppression may occur [62]. Intranasal budesonide irrigations given for a period of at least 1 month do not appear to increase intraocular pressure [63]. In-office treatment with a steroid-eluting implant with mometasone furoate significantly reduces nasal congestion score, bilateral polyp grade and ethmoid sinus obstruction in a group of CRS patients with recurrent polyposis after ESS [64,65,66]. An implantable matrix that locally elutes mometasone furoate has shown to be effective, safe and well tolerated over the 24-week treatment period [67]. Perhaps in the future supramolecular materials loaded with steroids will be widely used in CRS [68].

#### 4.1.3. Systemic Steroids

Oral corticosteroids (OCS) should be used only in the short-term (7–21 days) due to different, systemic adverse events. Short courses of OCS result in a significant reduction in symptom score and NP score and can be applied 1–2 times per year in patients with partially or uncontrolled CRS [2,69]. The effectiveness of this treatment has been demonstrated in many double blinded placebo-controlled trials [70,71,72,73,74,75]. Various doses of OCS, from 15 mg to 1 mg/kg daily, are used perioperatively and clinical trials are needed to determine the optimal dose [76]. Postoperative OCS as an add-on treatment for CRS should not be routinely recommended [77,78]. It was observed that a short course of OCS is more effective than INCS in eCRSsNP [79].

#### 4.1.4. Antibiotics

The EPOS steering group is uncertain whether or not the use of topical and intravenous antibiotics has an impact on patient outcomes in adults with CRS [2].

Using long-term, low-dose macrolide treatment could be a possibility for a selected group of patients with low-tissue and serum eosinophilia and unresponsiveness to corticosteroids [80,81], but it is uncertain whether or not the use of long-term antibiotics has an impact on patient outcomes [2]. Long-term, low-dose clarithromycin treatment is more effective in CRSsNP than in CRSwNP [82]. Low total IgE in the nasal secretion might predict low-dose macrolide response [83]. In studies with long-term azithromycin treatment, diverging results were obtained and further clinical trials are needed [84,85]. In a study assessing the effect of erythromycin in treatment of patients after surgery for CRS only the nasal endoscopy score showed a statistically significant improvement [86]. Cardiovascular risks of using macrolides should be always taken into account [2,87].

Using short-term oral antibiotics is not recommended as a standard therapy for adult CRS patients due to the very low-quality evidence of effectiveness [2]. Using amoxicillin-clavulanate for patients with acute exacerbation of CRS did not change the clinical course of this disease [88]. It has been suggested that short-term oral antibiotics poorly penetrate SM [89].

Use of local and systemic antifungal treatments in patients with CRS is not recommended [2].

### 4.2. Endoscopic Sinus Surgery (ESS)

In localized CRS, surgery should be performed as a first-line treatment. In diffuse CRS, surgery is indicated if the well-conducted pharmacological therapy is ineffective [2,13]. The main goal of ESS is improving the severity of symptoms, achieving drainage and ventilation of the sinuses, and also decreasing inflammatory load, removing eosinophils and polyps. Recently a new surgical technique (reboot approach) has been developed which aims to completely remove all diseased SM and allow healthy re-epithelialization from the preserved SM. This technique used in type 2 inflammatory CRSwNP significantly reduces the recurrence of NPs postoperatively [90]. Steroid-eluting sinus implants have been shown in clinical trials to improve postoperative outcomes after ESS [91,92,93,94,95,96]. Preoperative administration of OCS improves the perioperative visibility by reducing blood loss and shortens the operation time [71]. Maintenance therapy with topical steroid after ESS is indicated in CRSwNP in order to provide better control of symptoms and to reduce the risk of polyp recurrence [2]. Use of intranasal gel with a bacteriophages mixture in a group of CRSwNP patients after ESS causes a decrease in the total number of microorganisms and also in the activity of secretory IL-1β and IL-8 [97].

### 4.3. Additional Anti-Inflammatory Treatment Methods in N-ERD Patients

(NSAID)-exacerbated respiratory disease (N-ERD) is a chronic eosinophilic, inflammatory disorder that consists of three conditions: asthma, recurrent NPs, and sensitivity to NSAIDs. N-ERD as a subtype of CRSwNP can be distinguished from this group by elevation in type 2 cytokines and IFN-γ, but among patients with N-ERD, a variability in inflammatory signatures is observed, an aspect which is important to clarify in consideration of the possibility to use biologics [98,99]. The major rule in management of N-ERD is avoidance of NSAIDs and cross-reactive drugs. Moreover, CRS in N-ERD should be treated similarly to eCRS, but also additional specific treatment options such as aspirin treatment after aspirin desensitization (ATAD), leukotriene-modifier drugs (LTMDs) can be used [2,53].

#### 4.3.1. Low Salicylate Diet

There have been reports that a low salicylate diet significantly improves sinonasal symptoms, QoL and endoscopic scores [100,101,102], but more research is needed. For N-ERD patients, avoiding alcohol should be recommended [53].

#### 4.3.2. Aspirin Treatment after Aspirin Desensitization

Aspirin treatment after aspirin desensitization (ATAD) with oral aspirin improves QoL, sinonasal symptoms, and forced expiratory volume in one second in N-ERD patients [2,99,103,104,105,106,107]. During this therapy, patients are firstly desensitized by exposure to increasing doses of oral aspirin and then continue using daily high doses of aspirin [103]. The majority of patients tolerate ATAD without any major complications [99]. It has to be emphasized that this method of treatment requires very good patient compliance. A retrospective multicentre study shows the high discontinuation rate of ATAD (63%) [108].

#### 4.3.3. Leukotriene Modifying Agents

Leukotrienes are synthesized by eosinophils and mast cells and play an important role in chronic inflammation through mucous production and bronchoconstriction. The number of cells expressing the cysteinyl leukotriene receptor 1 in nasal tissue is significantly higher in the aspirin sensitive patients compared to non-aspirin sensitive patients in CRSwNP [109]. LTMDs are anti-inflammatory drugs used for many years in asthma. There are two types of LTMDs: leukotriene receptor antagonists (montelukast, zafirlukast, pranlukast) and 5-lipoxygenase inhibitors (zileuton). In the case of CRS, the use of montelukast should be limited to the patients with INCS intolerance due to the lack of benefit over the use of INCS or when added to INCS [2,110]. LTMDs have moderate effects in relieving nasal symptoms and NPs size in N-ERD patients, but are not more effective in N-ERD compared to NSAIDs-tolerant CRSwNP patients [99]. The efficiency of postoperative montelukast therapy in patients with N-ERD has been demonstrated to reduce NP recurrence and positive effects on sinonasal symptoms [111]. Zileuton in the treatment of N-ERD causes no improvement in rhinologic QoL symptoms but may be useful in diminishing frequency of surgical intervention [112,113]. N-ERD patients with both CRS and asthma might benefit from LTMD, but the indication should be derived from the lower airway disease status [103].

## 5. New Emerging Immunomodulating Therapeutic Options—Biologics

Monoclonal antibodies (MAb) are biologics used in oncological diseases, asthma, psoriasis, atopic dermatitis and other disorders where immune system dysfunctions are noticed. Due to the common Th-2 immune pathway, most of the biologics developed for asthma are effective in CRSwNP. Biologic treatment can be used in CRSwNP patients meeting the eligibility criteria (Table 1) [2].

Criteria for the efficiency assessment of biologics are shown in Table 2 [2]. The randomized, placebo-controlled trials conducted so far concerned MAb directed against the specific type 2 immune response targets (IL-4, IL-5 or free IgE) (Figure 3). In 2019, dupilumab became the first biologic approved by the Food and Drug Administration (FDA) and the European Medicines Agency (EMA) for adult patients with inadequately controlled CRSwNP. However, so far biologics are not on the list of reimbursed medicines in all countries. Unfortunately, the costs of this treatment without support are unacceptable by the majority of patients.

### 5.1. Anti-IgE Antibodies

Omalizumab is a recombinant humanized Mab (HuMAb) that selectively binds to human IgE and prevents the binding of free IgE to the high-affinity IgE receptor (FcεRI) on basophils and mast cells. The result is a decreased level of free serum IgE and consequently the blocking of the IgE-mediated inflammatory cascade [114]. In 2020, omalizumab has been approved by FDA and EMA as an add-on maintenance treatment with INCS for adults with severe CRSwNP. This drug is indicated in moderate to severe asthma in patients and in chronic idiopathic urticaria. Omalizumab is administered subcutaneously every two or four weeks. The optimal dosage depends on baseline serum total IgE level and patient weight. Omalizumab added to INCS therapy resulted in beneficial effects on nasal symptoms (nasal congestion, anterior rhinorrhoea, loss of smell, wheezing, dyspnoea) and QoL scores, alongside reduced polyp sizes [115,116]. After omalizumab discontinuation, scores gradually worsened over the 24-week follow-up, but remained improved from pre-treatment levels [117]. Omalizumab is characterized by a good safety profile. The most common adverse reactions were: headache, dizziness, arthralgia, abdominal pain and injection site reactions. Patients with moderate to severe asthma receiving omalizumab demonstrated a higher incidence rate of cardiovascular and cerebrovascular events [118]. Currently, novel anti-IgE antibodies such ligelizumab, quilizumab or MEDI4212 are being tested in various disorders [119].

### 5.2. Anti-IL-4Rα Antibodies

Dupilumab is a fully HuMAb that inhibits type 2 inflammation through blocking the shared receptor component for IL-4 and IL-13—IL-4 receptor α chain (IL-4Rα), resulting in blocking those two cytokine signalling pathways through the STAT6 pathway. Currently, dupilumab is indicated as being added to the daily standard of care in adult patients with uncontrolled CRSwNP [2] and is also used in other conditions characterized by local and systemic eosinophilia such as atopic dermatitis and asthma. Dupilumab is available in prefilled syringes (300 mg) and is administered subcutaneously every two weeks in addition to standard treatment. Dupilumab added to INCS therapy reduced severity of symptoms, endoscopic polyp size, sinus opacification, markers of Th2 inflammation and, in patients with coexisting asthma, improved pulmonary function (forced expiratory volume in one second) and control of asthma symptoms [120,121,122,123,124,125,126,127]. Dupilumab also reduced the number of patients undergoing ESS and the need for OCS use [128]. In studies presenting outcomes of dupilumab therapy in real-word settings, the efficiency and safety of this treatment in severe uncontrolled CRSwNP were proved [129,130,131,132]. Dupilumab is a safe medicine, causing usually mild side effects such as: nasopharyngitis, injection site reactions, epistaxis, and headache. Cases of eosinophilia with clinical symptoms were observed during treatment: eosinophilic granulomatosis with polyangiitis (EGPA), eosinophilia associated with arthralgia, asthma exacerbation, and insomnia [125]. After assessment across 11 dupilumab clinical trials, it was conducted that transient increases in eosinophil counts with dupilumab treatment did not affect efficiency and were rarely of clinical consequence [133].

### 5.3. Biologics Targeting IL-5

#### 5.3.1. Drugs Attaching to Free IL-5 and Blocking Its Activity

Reslizumab and mepolizumab are humanized antibodies that bind to free IL-5, preventing it from binding to the IL-5 receptor α chain (IL-5Rα) on eosinophils and basophils, which cause impairing eosinophilic inflammation.

Reslizumab is administered intravenously every four weeks and registered for the treatment of severe, uncontrolled, eosinophilic asthma in addition to other asthma medicines. Gevaert’s study on CRSwNP patients, in which a single intravenous infusion of reslizumab was administered confirmed that NP scores improved only in half of the patients and increased nasal IL-5 levels predicted the response to anti-IL-5 treatment [134]. Reslizumab has a good safety profile [135].

Mepolizumab is administered subcutaneously every four weeks and registered in eosinophilic asthma, severe CRSwNP, EGPA, and hypereosinophilic syndrome. In two studies on patients with CRSwNP, mepolizumab added to daily INCS treatment led to a reduction in the need for ESS, improved sinonasal symptoms, endoscopic polyp size, and sinus opacification [136,137,138]. Mepolizumab reduces SM eosinophils, but concurrently can lead to a local type-2 inflammatory feedback loop [139]. Mepolizumab is characterized by a good safety profile. The most common adverse reactions were headache, injection site reactions and back pain.

#### 5.3.2. Biologic Targeting IL-5 Receptor

Benralizumab is a HuMAb targeting the α chain of the IL-5 receptor (IL-5Rα). Currently, benralizumab is indicated for patients with severe, eosinophilic asthma in addition to other asthma medicines. Benralizumab is injected subcutaneously every 4 weeks for the first 3 doses and every 8 weeks afterwards. Studies on patients with CRSwNP demonstrated that benralizumab added to INCS reduced nasal blockage, difficulty with sense of smell, endoscopic polyp size, sinus opacification, but some of the results were not statistically significant [140,141,142]. Benralizumab has a good safety profile.

### 5.4. Biological Treatment Strategy and Perspectives

Further studies with large groups of patients are necessary to compare the efficiency and safety of individual biologics in different groups of patients, because it is still unclear which biologic agent should be used for which patient. On the basis of currently available information, dupilumab may be the best choice for CRSwNP treatment when compared with omalizumab, mepolizumab, and benralizumab at 24 weeks of the treatment [143]. The issue of different enrolment criteria in clinical trials for biologics should also be mentioned, because comparable patient populations and standardized outcome measures would help to compare effects of different biologic agents [144]. The most reliable information would provide direct randomized trials with the use of different biologics.

It is still unknown whether the treatment can prevent the development of CRS and how the reaction to the biologic drugs can be predicted. There is also a need for studies combining surgery and biological treatment to assess whether and at what moment (in pre or postoperative period) biologics should be added. Biologics used in CRS are considered to be safe, but there is lack of research informing the long-term effects. It has not been established how long biological drugs should be taken—lifelong or in sporadic courses. Finally, there remains the question of the cost-effectiveness of biologics compared to the traditional methods of treatment, including ESS.

## 6. Potential Novel Targets in CRS Immunotherapy

The following are some chosen examples of drugs which could be potentially used in CRS, but studies conducted so far are insufficient to determine their role and their usage in CRS.

### 6.1. TSLP Inhibitors

#### 6.1.1. Tezepelumab

Tezepelumab is a HuMAb that inhibits the action of thymic stromal lymphopoietin (TSLP)—an epithelial cell-derived cytokine that is implicated in the pathogenesis of CRS. TSLP interacts directly with mast cells to promote eosinophilic inflammation through the production of Th2-type cytokines and is involved in both T2 and non-T2 inflammation [145]. Tezepelumab is administered subcutaneously once every four weeks and indicated in severe, uncontrolled asthma with no phenotype or biomarker limitations as an add-on maintenance treatment. Until the present day there are no results of clinical trials of tezepelumab in CRS. A multicentre study on tezepelumab in severe CRSwNP (WAYPOINT) is in progress and estimated study completion is in 2024. Currently, there are available results from randomized studies evaluating the efficiency and safety of tezepelumab in asthma and atopic dermatitis.

#### 6.1.2. Other TSLP Inhibitors under Clinical Development

ASP7266 is a novel HuMAb against Human Thymic Stromal Lymphopoietin Receptor (TSLPR). ASP7266 exhibited excellent pharmacological activity in preclinical studies and has the potential to be a promising treatment option for patients with allergic disorders, including eCRS [146]. There are other biologic agents targeting TSLP (MK-8226, RG7258, CSJ117), but trials were discontinued or results are unavailable [147].

### 6.2. Anti-IL-33

IL-33, a member of the IL-1-family, is one of the epithelial cell-derived cytokines that binds to a heterodimeric cell-surface receptor consisting of an IL-1 receptor accessory protein and ST2 on Th2 cells, ILC2s, basophils, eosinophils, mast cells, dendritic cells, neutrophils, regulatory T cells, macrophages and others [2,148]. Tissue damage is a nonspecific trigger of epithelial IL-33 production in treatment-recalcitrant polyps [149]. The potential role of IL-33 in the pathogenesis of CRSwNP is through neutrophil recruitment [150]. IL-33 and its receptor may be a target in CRS treatment in the future. Etokimab and AMG 282 are biologics targeting IL-33 that have been already investigated to evaluate their efficiency and safety in CRSwNP, but detailed results of these studies are not yet published. Etokimab is a HuMAb with a good safety profile that has a high affinity to human IL-33 and inhibits its activity [151]. AMG 282 is an anti-ST2 Mab that inhibits the binding of IL-33 to the ST2 receptor [152]. Other anti-IL-33 drugs (GSK3772847, SAR440340) are already tested in asthma, COPD and other diseases.

### 6.3. Anti-IL-25

IL-25 is an epithelial cell-derived cytokine of the IL-17 family involved in innate and adaptive pathways in immunopathology of CRS. IL-25 is overexpressed in NP tissue from Asian patients with CRSwNP [153,154]. There is a chance that IL-25 can be a potential therapeutic target in CRS.

### 6.4. Anti-Siglec-8

Sialic-acid-binding immunoglobulin-like lectin (Siglec)-8 is a receptor selectively expressed on human eosinophils, basophils, and mast cells. Siglec 8 cross-linking with antibodies results in induction of eosinophil apoptosis [155] and also inhibition of mast cell degranulation [156]. AK001 targeting siglec-8 has been tested in CRSwNP, but the study was stopped earlier and resulted in uninterpretable data (unpublished). Now, the efficiency of another anti-siglec-8 antibody lirentelimab (known also as AK002) is tested in eosinophilic esophagitis, gastritis, gastroenteritis, duodenitis, conjunctivitis, chronic urticaria, atopic dermatitis, and actinic keratosis. Siglec-8 seems to be a promising therapeutic target in eosinophilic inflammatory diseases, hopefully also in CRS.

### 6.5. GATA-3 Specific DNAzyme

The transcription factor GATA-3 controls the expression and production of IL-3, IL-5, and IL-13, which is essential for type 2 inflammation and is overexpressed in patients with CRSwNP, asthma, and atopic eczema [157]. Inhibition of GATA-3 is possible by using DNA molecules that bind specific GATA-3 sequences, an example of which is SB010, administered by inhalation, that has been already tested in eosinophilic chronic obstructive pulmonary disease and asthma and promising results have been reported [158,159]. So far, no studies with GATA-3 DNAzyme have been performed in CRS, but there is an opportunity that intranasal drugs targeting GATA-3 may be beneficial in this disease.

### 6.6. Anti-TNFα

TNFα is a key cytokine in endotoxin induced neutrophilic inflammation and theoretically could be a potential target for treatment of CRS, because it inhibits endotoxin-induced neutrophilic inflammation [160]. It has been demonstrated that TNFα is the influencing factor of refractory CRS [161]. Currently, anti-TNFα treatment is already used in many immune-mediated inflammatory diseases but has not been tested in CRS. Wand et al. noticed that the application of this therapy can be associated with new-onset sinusitis, mainly CRSsNP [162].

### 6.7. Anti-IL-17A

IL-17 is widely known as playing a crucial role in type 3 immune response in CRSsNP, but the potential role of IL-17 in local neutrophilic inflammation in NPs has been also demonstrated [163,164]. Furthermore, it is considered that IL-17 may affect the prognosis of CRSwNP [165]. Using IL-17 inhibitors in both endotypes of CRS seems to be rational. Nevertheless, antibodies that inhibit the biological activity of IL-17A have been already tested in psoriasis, arthritis, asthma and Crohn’s disease, but not in CRS.

### 6.8. Bispecific Antibodies

Venkataramani et al. designed and developed a novel highly potent bispecific antibody against two attractive targets in type 2 immune response—TSLP and IL-13, Zweimabs and Doppelmabs, that concurrently inhibit the signalling by two cytokines. It was thus shown that dual targeting is possible [166]. Maybe this type of biologic agent will prove useful in CRS in the future.

## 7. Conclusions

A better understanding of immune mechanisms involved in each CRS endotype gives hope for developing effective diagnostics and therapeutic solutions tailored for each individual patient. The classification based on the division into endotypes, that reflect the pathogenesis of CRS, allows for the isolation of groups of patients who could receive targeted therapy with the use of antibodies. Various biologics have been confirmed to be effective as add-on treatments to INCS and those drugs are particularly important in the therapy of patients with recurrent polyps, resistant to conventional methods of treatment. Hopefully, effective therapeutic solutions for every endotype will be widely available very soon.

**Take-Home Messages:**

- CRS is a multifactorial, heterogeneous group of diseases, in which frequently conventional therapy is insufficient.
- Type 2 inflammation plays a key role in eosinophilic inflammation in CRSwNP.
- Various cytokines and cytokine receptors can be targets for biological treatment in CRSwNP resistant to conventional treatment.
- Future studies are needed to establish biomarkers to identify endotypes and predict response to treatment with biologics in CRSwNP.

## Figures and Tables

**Table 1:** Indications for biological treatment in CRSwNP according EPOS 2020 [2] (ESS—endoscopic sinus surgery, QoL—quality of life).

Indications for Biological Treatment in CRSwNP
**patients with bilateral polyps who underwent ESS** (or have contraindications to surgery)
and meet min. 3 criteria of the following:
→ evidence of type 2 inflammation (tissue eosinophilia ≥ 10/hpf, or blood eosinophilia ≥ 250, or total IgE ≥ 100)
→ need for systemic corticosteroids or contraindication to systemic steroids
→ significantly impaired QoL
→ significant loss of smell
→ diagnosis of comorbid asthma

**Table 2:** Criteria for the efficiency assessment of biologic therapy according EPOS 2020 [2] (NPs—nasal polyps, QoL—quality of life).

Criteria for the Efficiency Assessment of Biologic Therapy (First Evaluation Should Be Carried out after 16 Weeks)
→ reduced NP size
→ reduced need for systemic corticosteroids
→ improved QoL
→ improved sense of smell
→ reduced impact of comorbidities

## Data Availability

Not applicable.
